# A systematic review of data sources for artificial intelligence applications in pediatric brain tumors in Europe: implications for bias and generalizability

**DOI:** 10.3389/fonc.2023.1285775

**Published:** 2023-10-27

**Authors:** Alberto Eugenio Tozzi, Ileana Croci, Paul Voicu, Francesco Dotta, Giovanna Stefania Colafati, Andrea Carai, Francesco Fabozzi, Giuseppe Lacanna, Roberto Premuselli, Angela Mastronuzzi

**Affiliations:** ^1^ Predictive and Preventive Medicine Research Unit, Bambino Gesù Children’s Hospital, IRCCS, Rome, Italy; ^2^ Department of Neuroscience and Imaging, “SS Annunziata” Hospital, “G. D’Annunzio” University, Chieti, Italy; ^3^ Imaging Department, Bambino Gesù Children’s Hospital, IRCCS, Rome, Italy; ^4^ Department of Neurosciences, Bambino Gesù Children’s Hospital, IRCCS, Rome, Italy; ^5^ Department of Hematology/Oncology, Cell and Gene Therapy, Bambino Gesù Children’s Hospital, IRCCS, Rome, Italy

**Keywords:** artificial intelligence, CNS tumors, pediatric oncology, childhood cancer, data sharing

## Abstract

**Introduction:**

Europe works to improve cancer management through the use of artificialintelligence (AI), and there is a need to accelerate the development of AI applications for childhood cancer. However, the current strategies used for algorithm development in childhood cancer may have bias and limited generalizability. This study reviewed existing publications on AI tools for pediatric brain tumors, Europe's most common type of childhood solid tumor, to examine the data sources for developing AI tools.

**Methods:**

We performed a bibliometric analysis of the publications on AI tools for pediatric brain tumors, and we examined the type of data used, data sources, and geographic location of cohorts to evaluate the generalizability of the algorithms.

**Results:**

We screened 10503 publications, and we selected 45. A total of 34/45 publications developing AI tools focused on glial tumors, while 35/45 used MRI as a source of information to predict the classification and prognosis. The median number of patients for algorithm development was 89 for single-center studies and 120 for multicenter studies. A total of 17/45 publications used pediatric datasets from the UK.

**Discussion:**

Since the development of AI tools for pediatric brain tumors is still in its infancy, there is a need to support data exchange and collaboration between centers to increase the number of patients used for algorithm training and improve their generalizability. To this end, there is a need for increased data exchange and collaboration between centers and to explore the applicability of decentralized privacy-preserving technologies consistent with the General Data Protection Regulation (GDPR). This is particularly important in light of using the European Health Data Space and international collaborations.

## 1 Introduction

AI holds the promise of addressing various unmet needs in cancer, although its systematic application is still in progress (1–3). A pivotal requirement for the successful integration of AI into healthcare is that data used to develop algorithms should be representative of the population’s diversity, ensuring the avoidance of bias and adverse patient events. Mitigating bias stemming from inadequate representation in the training of healthcare AI tools is a widely acknowledged challenge, demanding targeted strategies for its resolution (4). The cornerstone of these strategies lies in the collection and integration of disparate datasets from various institutions, thereby circumventing the underrepresentation of specific population subsets and the potential ensuing discrimination. Numerous global initiatives have been launched to create openly accessible datasets that encompass a variety of data types essential for training and validating AI systems (5). Concurrently, the feasibility of consolidating datasets from multiple European centers is subject to adhering to GDPR regulations, which could potentially hamper data sharing for scientific purposes (6, 7). Within this context, a notable concern arises: how the current AI tools customized for clinical use in Europe are trained with datasets representing diverse populations.

In 2021, the European Commission introduced the Europe’s Beating Cancer Plan, a strategic framework aimed at bridging gaps in cancer prevention, treatment, and care, with a specific emphasis on childhood cancer priorities (8). This plan notably aligns with the European Digital Strategy’s endorsement of Artificial Intelligence (9). Building on this perspective, the European Society for Pediatric Oncology envisions harnessing AI technologies to their full potential for the benefit of pediatric cancer patients in Europe (10).

Central nervous system (CNS) tumors constitute nearly 20% of childhood cancers, making them the predominant solid neoplasms in this age group (11). Despite significant progress that has improved the outlook for pediatric CNS tumor patients, challenges persist in fully implementing precision medicine, enabling less invasive interventions, predicting treatment responses, and identifying new therapeutic approaches (10–12).

Childhood CNS tumors significantly differ from their adult counterparts by incidence, histology, molecular biology, treatment strategies, outcomes, and long-term outcomes. Consequently, extrapolating data from adults to children is not appropriate (13). Conversely, the rarity of brain tumors in pediatric patients poses a challenge in amassing a substantial volume of observations to appropriately train algorithms in this disease group.

The WHO stresses the need for AI developers to ensure that AI data is accurate, comprehensive, and representative of diverse age groups, including children (14, 15). Moreover, the proposed EU AI Act requires that “…training, validation and testing data sets shall be relevant, representative, free of errors and complete” (Art. 10) (16). In fact, a substantial portion of existing research on childhood CNS tumors consists of proof-of-concept studies based on limited datasets, which makes them not yet directly applicable into clinical practice (12, 17).

All this considered, we undertook a systematic review of the current literature related to AI tools specifically developed for tackling pediatric brain tumors in the European context. Our objective was to describe the characteristics of the data utilized in their development and the potential associated bias. Our inquiry centered on delineating the sources of data, gauging the scale of the datasets, evaluating their interoperability, ascertaining the presence of external validation, and scrutinizing the geographic representation of the cohorts employed in refining the algorithms. Based on these findings, we discuss potential strategies to accelerate the development and integration of AI tools for pediatric brain tumors into clinical practice in Europe.

## 2 Methods

We performed a systematic review of the current literature by employing a search query based on the terms recommended by the Cochrane collaboration for pediatric tumors (18), including terms specific to pediatric brain cancer. Furthermore, we developed a distinct search query tailored to encompass AI techniques, subsequently merging it with the prior query (**Supplemental materials - S1**).

Our search spanned the databases of MEDLINE, EMBASE, Web of Science, and Scopus, limiting the inquiry to papers published in English within the period spanning January 2010 to May 31, 2022. The outcomes of this search were imported into the Rayyan software (19) and subjected to a duplicate screening process.

Subsequent to this screening, we manually reviewed the remaining records to ascertain their eligibility against a set of predefined criteria, encompassing: 1) original articles; 2) papers detailing the development of AI tools tailored for pediatric brain tumor diagnosis, prognosis prediction, or therapeutic decision support; 3) at least one author affiliated with a European institution; 4) publications in English. Conversely, we excluded articles: 1) not presenting original data or reviews; 2) published in languages other than English; 3) authored by individuals not affiliated with European institutions; 4) describing studies conducted on animals or simulated environments.

We have included authors from institutions based in the UK, as they continue to be eligible for collaborative projects within established European networks following Brexit.

The selected articles were categorized into two distinct groups: 1) those exclusively comprising observations from patients under 18 years of age for algorithm training; 2) those encompassing both pediatric and adult populations.

Bibliometric details were extracted in a format compatible with bibliometric analytical tools, while three independent reviewers manually extracted specific details from each publication, including: a) the specific brain tumor type under examination; b) the scope of the AI tool; c) the nature of data utilized; d) the data repository employed; e) the count of patients contributing data for algorithm development; e) the use of data standards for interoperability; f) the use of external validation of the algorithms; g) the performance of the AI tool; h) the geographic representation of the cohorts used for developing the algorithm.

The resulting information was subjected to descriptive analysis, with bibliometric data analyzed through the Biblioshiny software (20). Additionally, we evaluated the FAIR Guiding Principles score (21) for each dataset included in the reviewed articles using the SATIFYD online tool (https://satifyd.dans.knaw.nl/). Other statistical analyses were performed utilizing the R software (22).

## 3 Results

**Figure 1:**
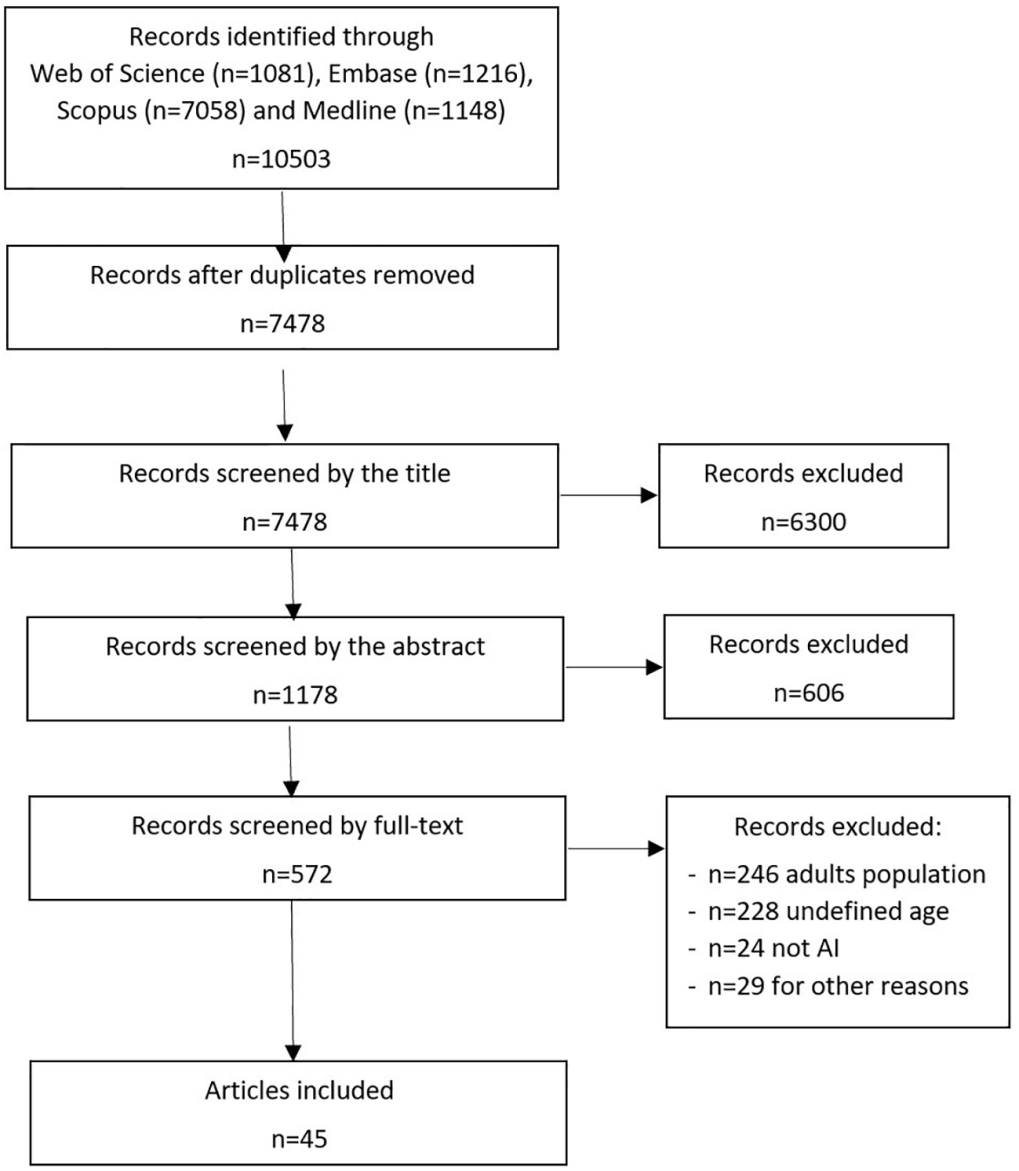
Review process of selected publications according to PRISMA.

Our search strategy yielded a total of 10503 scientific publications. The selection process adhered to PRISMA standards and is visually represented in **Figure 1**. Upon eliminating duplicates across diverse databases, we identified 7478 unique records. Subsequent review of titles and abstracts narrowed down the selection to 572 records, whose full texts were evaluated. Within this group, we excluded 246 articles solely centered on adult populations, 24 that didn’t involve AI methodologies, and 29 that didn’t meet the eligibility criteria for various reasons. This left us with 273 remaining studies.

Of these 273 studies, 228 failed to report the age of the patients and were consequently excluded. Ultimately, we selected 45 articles that incorporated pediatric data for the AI algorithm development. Among these, 25 articles exclusively focused on children, while 20 articles used data from both pediatric and adult cohorts (**Supplemental materials - S2**).

Our review encompassed publications dating back to 2012. Notably, 27 out of the 45 articles (60.0%) included in this study were published from 2020 onward. Additionally, eight studies were published before 2016 and were characterized by relatively modest patient numbers during algorithm training, employing simple AI techniques. The array of AI methods utilized for analysis was diverse, encompassing techniques such as LDA, KNN, Naive Bayes, SVM, Random Forest, and more. Similarly, the choice of software varied, including Python, R, and Mazda Orange, although the preponderance of papers analyzed data within a Python environment. On average, the selected publications garnered 57.8 citations each.

**Table 1** provides an overview of the brain tumor types for which AI algorithms were developed, categorized by the included population type. The majority of publications focused their efforts on glial tumors, while other tumor types were addressed less frequently. The category of unclassified tumors encompassed those specifically characterized as brain or posterior fossa tumors, pituitary adenoma, and large B-cell lymphoma. **Table 2** shows the scope of the articles included in this review by type of population included. Notably, the predominant thrust of the selected papers (24 out of 45, accounting for 53.3%) was directed towards algorithmic development for tumor classification. Conversely, fewer papers pertained to prognosis, decision support, or validation tasks.

**Table 1:** Diagnoses included in the selected publications by type of population.

	Pediatric population only (n=25)	Children and adults (n=20)	Total (n=45)
	n (%)	n (%)	n (%)
Glial tumors	20 (80.0%)	14 (70.0%)	34 (75.5%)
PNET	19 (76.0%)	–	19 (42.2%)
Ependymal tumors	18 (72.0%)	-	18 (40.0%)
Mesenchymal tumors	1 (4.0%)	1 (5.0%)	2 (4.4%)
Germ cell tumors	1 (4.0%)	–	1 (2.2%)
Unclassified	4 (16.0%)	6 (30.0%)	10 (22.2%)

Multiple diagnoses may be included in the same publications.

**Table 2:** Scopes of the articles included in the review.

	Pediatric population only (n=25)	Children and adults (n=20)	Total (n=45)
	n (%)	n (%)	n (%)
Classification	17 (68.0%)	9 (45.0%)	26 (57.8%)
Diagnosis	6 (24.0%)	3 (15.0%)	9 (20.0%)
Prognosis	3 (12.0%)	8 (40.0%)	11 (24.4%)
Therapy	1 (4.0%)	1 (5.0%)	2 (4.4%)

Multiple scopes may be included in the same publications.

The majority of publications employed the same dataset for both testing and validating their algorithms, with only 3 out of 45 utilizing external datasets for validation. When reported, the external validation performance of the algorithm was lower than that reported for internal validation.

**Table 3** shows the data sources utilized across the reviewed publications. The majority of AI studies examined diagnostic images as primary data sources, specifically employing multiple conventional MRI sequences for classification and prognosis of CNS tumors. However, some studies delved into spectroscopy, a more specialized MRI sequence often utilized at the point of diagnosis (23). Additionally, computer tomography and histopathology images made appearances as data sources. While metabolite profiles, epigenetics, gene expression, and clinical features were used less frequently than images, only 6 out of 45 publications (13.3%) integrated data from multiple sources.

**Table 3:** Data sources used in the selected publications by type of population.

	Pediatric population only (n=25)	Children and adults (n=20)	Total (n=45)
	n (%)	n (%)	n (%)
MRI	24 (96.0%)	11 (55.0%)	35 (77.8%)
Histopathology	1 (4.0%)	2 (10.0%)	3 (6.7%)
DNA methylation	1 (4.0%)	3 (15.0%)	4 (8.9%)
CT	1 (4.0%)	–	1 (2.2%)
Metabolite profiles	2 (8.0%)	–	2 (4.4%)
Gene expression	–	1 (5.0%)	1 (2.2%)
Other	1 (4.0%)	2 (10%)	3 (6.7%)

Multiple data sources may be included in the same publications.

**Table 4** shows the repositories gathering data from multiple sites used for the development of algorithms and accessible on the web, as reported in the articles included in the review.

**Table 4:** Data repositories gathering data from multiple sites used in articles included in this review.

Name	Type of data	URL	Population	Type of access
Children’s Cancer and Leukaemia Group - Functional Imaging Database	Functional Imaging of Tumours study with CCLG from a UK network of hospitals.	https://www.cclg.org.uk/	Children	Restricted
eTUMOUR*	MRI, SV 1H MRS data and histology of brain tumors	http://www.etumour.net/	Children	Restricted
Gene Expression Omnibus	Functional genomics data repository	https://www.ncbi.nlm.nih.gov/geo/	All ages	Public, open
The Cancer Genome Atlas	Genomic, epigenomic, transcriptomic, and proteomic data of patients with cancer	https://www.cancer.gov/ccg/research/genome-sequencing/tcga	All ages	Public, open
The Cancer Imaging Archive	Imaging, patient outcomes, treatment details, genomics, pathology of patients with cancer	https://www.cancerimagingarchive.net/	All ages	Public, open
Belgian Cancer Registry	Registry of newly diagnosed cases of cancer in Belgium	http://kankerregister.org/Home_en	All ages	Restricted
BraTS2020	MRI images and clinical information from patients with brain tumors	https://www.med.upenn.edu/cbica/brats2020/data.html	All ages	Restricted

*This repository was developed during a project funded by the European Commission, and it is no longer accessible.

Considering data origin, 29 articles (64.4%) drew from single centers, while 16 (35.6%) were the result of multicenter collaborations. Two studies used synthetic data to inform their algorithms’ development, and no instances of federated learning were noted. Notably, no selected publication provided insights into the interoperability of their datasets.

As for the FAIR Guiding Principles score, the median value for the datasets used in the articles in this review stood at 23 (IQR: 15-32). This relatively modest score stemmed from the frequent absence of accessible metadata and from the limited accessibility of datasets used for algorithm development.

The number of patients included in the datasets for the development of AI algorithms was quite variable and essentially limited for most data sources. The median observation count used for single-center studies was 89, whereas publications emerging from multicenter collaborations exhibited a median observation count of 120.

Examining geographic scope, 7 out of the 45 reviewed articles tapped into public repositories for data. An additional 14 articles incorporated data from beyond the EU, including countries such as the USA, Canada, China, Iran, and Argentina. In terms of pediatric datasets within the EU, 17 publications (37.8%) harnessed cohorts from the UK, while individual datasets emerged from Belgium, Denmark, Italy, Netherlands, Spain, and Sweden.

**Figure 2:**
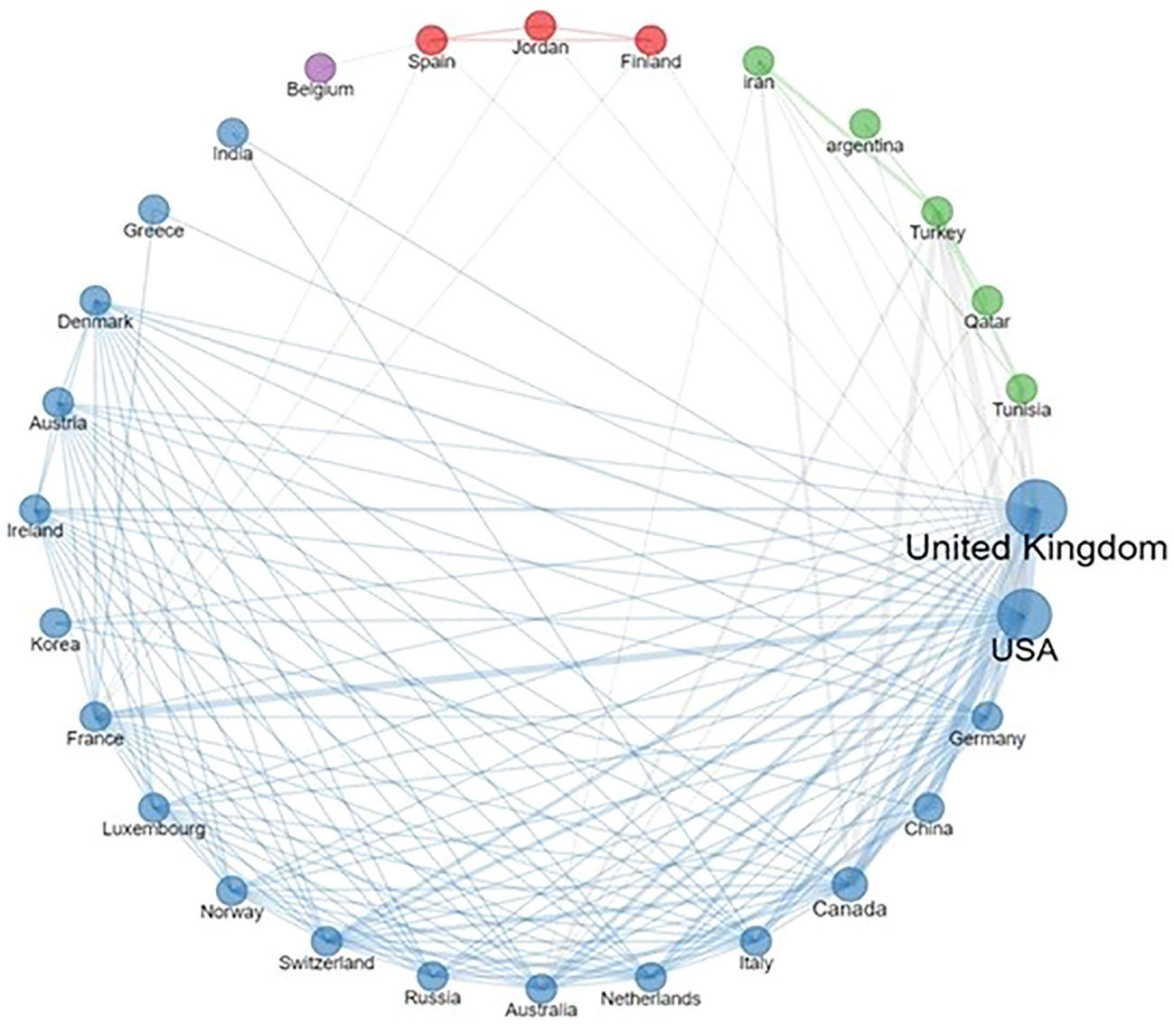
Country collaboration network. The thickness of lines reflects the frequency of collaborations.

Delving into the collaborative landscape, **Figure 2** illustrates the interconnected network of institutions represented across the selected publications. Notably, the UK stood out as a frequent contributor, not just in terms of author affiliations, but also due to its extensive collaborations with both European and non-European entities. Moreover, the network reveals consistent partnerships between EU countries and the USA.

## 4 Discussion

Our review unveiled a scarcity of publications regarding AI applied to CNS tumors in children in Europe. Most of the studies emerged from individual centers, indicating a pressing need for improving research in this field. This holds particular significance for the less prevalent tumor types, often overshadowed in the development of AI tools.

Furthermore, our review underscored that several European institutions are engaged in crafting AI tools for identical categories of pediatric brain tumors, with only 16 publications reflecting multicenter data contributions. On the other hand, we did not find any information about data standards for interoperability that can support collaborative research in this field. Notably, collaborative networks, both within Europe and on a global scale, emerged from our analysis, illustrating a fertile ground for reinforcing and expanding such partnerships.

We also observed that most publications reported AI tools trained with a low number of observations from selected populations. Moreover, few publications reported an external validation of their algorithms.

As in many other medical fields, research on AI on pediatric CNS tumors suffers from fragmented data and studies performed on small sample sizes. Although the performance of the published algorithm in this domain is fair according to reported results for internal validation, the potential for bias is high and their generalizability may be limited.

One of the major obstacles to data-sharing and collaboration among institutions in the development of AI tools that are trained on diverse and representative populations is the concern for privacy. This is particularly relevant in the EU, where the General Data Protection Regulation (GDPR) is in place. Data-sharing agreements require a thorough evaluation of potential risks, which can take a significant amount of time to complete. Additionally, there is still variability in the interpretation of GDPR requirements for data sharing at the country level (24, 25). Identifying the trade-off between privacy preservation and the full development of AI solutions will represent one of the most important topics for discussion at the data governance level.

While compliance with regulations for data sharing is important for scientific research in the field of AI (26), technologies such as federated and swarm learning (27–29) and synthetic data (30) may be prioritized to accelerate the development of AI tools. Notably, none of the publications reviewed in our study used federated or swarm learning and only two studies explored the use of synthetic data.

The datasets examined in the review seldom adhered to all FAIR principles, reinforcing the observation of a limited commitment to reusing and integrating data across different contexts.

Moreover, interoperability and data harmonization (31) are critical for addressing the fragmentation of data. The Observational Health Data Sciences and Informatics (OHDSI) community conducts research to promote the use of standards such as OMOP and standardized vocabularies (32, 33). Another standard that supports interoperability is HL7 FHIR (34). However, our review did not find any information on the interoperability of the datasets used for the development of AI tools.

The effectiveness of artificial intelligence tools is dependent on the quality of data they are trained on. With population selection, misrepresentation, or missing data, generalizability across subgroups is not possible. Our review found publications that used data from both children and adults for the development of algorithms for brain cancer, which may hinder their generalizability. The recent WHO document (13) on a detailed classification of CNS tumors in children provides a valuable tool for distinguishing different types on a molecular basis. AI tools can further improve classification if algorithms are trained with generalizable data.

The majority of AI tools used in the publications reviewed utilized diagnostic images as their data source. Neuroimaging, a well-developed area for AI in oncology, offers detailed analysis of brain microstructures and pathophysiology specific to children (35). However, AI also has the potential for integrating multiple types of data, which could be beneficial for investigating complex patterns and increasing algorithm accuracy. Our review found that only a small number of publications utilized this approach, which is an area that should be given more attention.

The development of AI tools outside medicine typically aggregates massive datasets from multiple sources which correlate with accurate predictions (36). Open repositories for developing AI tools represent an attempt to concentrate large amounts of diagnostic images and overcome the existing barriers to data access. In this review, among the 45 publications included, 7 have used a data repository for the development of their algorithm, of which 3 only were publicly accessible. Several public datasets are available, particularly for neuroimaging, which represents the main area of application of AI in neuro-oncology.

Unfortunately, public datasets for AI purposes are limited in size and do not accurately represent the entire population of children with brain tumors. Additionally, neuroimaging data in these datasets may have inconsistencies due to tumor and scan variations. Furthermore, advances in technology can lead to changes in the quality and features of diagnostic images, known as data drift, which can affect the performance of AI algorithms. This highlights the need for ongoing updates to the datasets used for training AI algorithms (37, 38).

In essence, the success of AI tools depends heavily on the quality and representativeness of the data used for training. Currently, many AI models for pediatric brain tumors are trained on small, single-center datasets. To enhance accuracy and applicability, there is a need for collaboration among research groups working on the same diseases, sharing data to enhance the robustness of AI models. Without this collective effort, AI models developed in isolation may lack the necessary accuracy for clinical decision-making, potentially introducing bias and errors. The absence of such collaboration leaves AI models vulnerable to overfitting, performing well on training data but poorly on external validation from different settings.

Our review has some limitations. Our study aimed to comprehensively review the existing literature on AI and pediatric brain tumor screening by using multiple bibliographic platforms and a structured search strategy with a manual review. However, information about the datasets used for developing AI tools may not be readily available in the literature, particularly if they are part of an industrial process. Therefore, policies that promote wider access and analysis of these datasets should be supported. Additionally, we intentionally focused on publications originating from the EU only. Our aim was to shed light on the challenges faced within the EU in terms of data sharing and AI model development taking into account factors as the General Data Protection Regulation (GDPR) and the issues related to data interoperability of the existing infrastructures. This approach is beneficial in understanding the European context, where there are many scientific collaborations and in light of the future creation of the European Health Data Space (39). Finally, we focused on data-driven bias only and we did not evaluate potential algorithmic bias in the studies included in this review.

In summary, this review highlights several potential sources of bias in AI tools developed for pediatric CNS tumors that may limit their clinical application. The most important strategy to address this limitation is promoting the use of larger and more diverse datasets through collaboration among different institutions that improve data availability, sharing, standardization, interoperability, continuous updates, and quality. On one hand, it is of paramount importance to promote the adoption of FAIR Principles during the development of AI tools. On the other hand, expanding existing networks that adhere to the same standards could benefit the development of EU-funded research and development projects, and data exchange through the European Health Data Space (39). Collaborative efforts should be supported by data standards such as those of Common Data Model (31, 32). Furthermore, decentralized privacy-preserving technologies such as federated learning and synthetic data may accelerate the development of AI tools based on large populations from different clinical sites while complying with EU privacy regulations (30, 40). Data access remains the biggest challenge in training and developing AI tools, and requires significant research effort and investment to enable the development of AI tools for pediatric brain tumors.

## Data availability statement

The original contributions presented in the study are included in the article/**Supplementary Material**. Further inquiries can be directed to the corresponding author.

## Author contributions

AT: Conceptualization, Methodology, Supervision, Writing – original draft, Writing – review & editing. IC: Data curation, Formal Analysis, Writing – original draft, Writing – review & editing. PV: Data curation, Formal Analysis, Writing – review & editing. FD: Data curation, Formal Analysis, Investigation, Writing – review & editing. GC: Data curation, Formal Analysis, Writing – review & editing. AC: Data curation, Supervision, Writing – review & editing. FF: Data curation, Formal Analysis, Investigation, Writing – review & editing. GL: Data curation, Formal Analysis, Writing – review & editing. RP: Supervision, Visualization, Writing – review & editing. AM: Conceptualization, Formal Analysis, Supervision, Writing – review & editing.
