# Supplementary material for: A systematic review of data sources for artificial intelligence applications in pediatric brain tumors in Europe: implications for bias and generalizability
Source: Front Oncol. 2023 Oct 27;13:1285775. doi: 10.3389/fonc.2023.1285775 (PMC10646175; doi:10.3389/fonc.2023.1285775)
Supplement: Supplementary file 1 [file Table_1.docx]

**Supplementary material**

**Search string:**

("artificial intelligence" OR "machine learning" OR "deep learning" "support vector machine" OR "random forest" OR "Markov decision process" OR "hidden Markov model" OR "fuzzy logic" OR "k-nearest neighbor" OR "naive Bayes" OR "Bayesian learning" OR "artificial neural network" OR "convolutional neural network" OR "recurrent neural network" OR "generative adversarial network" OR "deep belief network" OR "perceptron" OR "natural language processing" OR "natural language understanding" OR radiomic*)

AND

(leukemia OR leukemi* OR leukaemi* OR "childhood ALL" OR AML OR lymphoma OR lymphom* OR hodgkin OR hodgkin* OR T-cell OR B-cell OR non-hodgkin OR sarcoma OR sarcom* OR sarcoma, Ewing's OR Ewing* OR osteosarcoma OR osteosarcom* OR “wilms tumor” OR wilms* OR nephroblastom* OR neuroblastoma OR neuroblastom* OR rhabdomyosarcoma OR rhabdomyosarcom* OR teratoma OR teratom* OR hepatoma OR hepatom* OR hepatoblastoma OR hepatoblastom* OR PNET OR medulloblastoma OR medulloblastom* OR PNET* OR "neuroectodermal tumor" OR retinoblastoma OR retinoblastom* OR meningioma OR meningiom* OR glioma OR gliom* OR "pediatric oncology" OR "paediatric oncology" OR "childhood cancer" OR "childhood tumor" OR "childhood tumors" OR "brain tumor*" OR "brain tumour*" OR “brain neoplasms" OR "central nervous system neoplasm" OR "central nervous system neoplasms" OR "central nervous system tumor*" OR "central nervous system tumour*" OR "brain cancer*" OR "brain neoplasm*" OR "intracranial neoplasm*")
